# Clozapine in First Episode Psychosis: The best is delayed

**DOI:** 10.1192/j.eurpsy.2023.2253

**Published:** 2023-07-19

**Authors:** J. B. Rodrigues, A. P. João, C. Adão, P. Trindade

**Affiliations:** 1 Department of Psychiatry and Mental Health, Centro Hospitalar de Lisboa Ocidental, Lisboa, Portugal

## Abstract

**Introduction:**

Only two out of three patients diagnosed with First Episode Psychosis (FEP) achieve symptom remission after the administration of two different antipsychotics, illustrating the high prevalence of treatment-resistance in FEP. Of those, 84% are treatment-resistant since illness onset. Response to initial treatment is one of the main long-term illness course predictors. The only medication approved for treatment-resistant psychosis is clozapine and studies have demonstrated its superior antipsychotic effect among this drug class.

**Objectives:**

The aims of this communication are to describe a clinical case of FEP with symptom remission achieved only with clozapine and to review the literature regarding the pattern of clozapine use in FEP, the main difficulties of implementing it and its impact on the prognosis of this patients.

**Methods:**

Relevant data from the patient’s medical record were collected. Pubmed database was searched using the terms “clozapine” and “first episode psychosis”.

**Results:**

A 50 year old woman without previous contact with psychiatric services was taken to the emergency room following behavioural disorganisation and heteroagressive conduct. Poisoning and referential delusions, as well as alienation of personal action and elementary auditory hallucinations were found and the patient was admitted in the psychiatric unit. She began treatment with Aripiprazole without therapeutic benefit and a switch to Paliperidone was made, with the same result. Clozapine was then titrated to a dose of 100mg/day, with resolution of all the positive symptoms mentioned above and she acquired total insight to the disease and need for treatment, being discharged with a diagnosis of schizophrenia. 9 articles, all from 2017 onwards, were collected from the Pubmed database.

**Conclusions:**

There’s reluctance in prescribing clozapine in treatment-resistance FEP. This is evidenced by the mean number of antipsychotic prescribed before clozapine - 2.74 to 4.85 - as well as the delay on its prescription - 294 to 2447 days - and its prescription to only 16% in a cohort of patients with FEP. The main reasons for this hesitation are the serious, albeit rare, side-effects, such as agranulocytosis and myocarditis, as well as the difficulty in implementing it in community services, with mandatory weekly blood tests and very slow titration of the drug and treatment compliance issues, making it a very resource-consuming drug. However, in that same cohort, there was a significant reduction of the number of admissions, re-admissions and duration of hospitalisation, highlighting the need for earlier consideration in treatment-resistant FEP. The identification of treatment-resistance should then be proactive by the mental-health services, ensuring an earlier clozapine initiation with the goal of greatly improving the prognosis of these patients

**Disclosure of Interest:**

None Declared

